# High-protein and low-calorie diets improved the anti-aging Klotho protein in the rats’ brain: the toxic role of high-fat diet

**DOI:** 10.1186/s12986-020-00508-1

**Published:** 2020-10-15

**Authors:** Anahid Shafie, Ahmad Mustafa Rahimi, Iraj Ahmadi, Fatemeh Nabavizadeh, Mina Ranjbaran, Ghorbangol Ashabi

**Affiliations:** 1 Department of Physiology, School of Medicine, Tehran University of Medical Sciences, P.O.box: 1417613151, Tehran, Iran; 2 Department of Physiology, School of Medicine, Ilam University of Medical Sciences, Ilam, Iran; 3 Department of Physiology, School of Medicine, Alberoni University, Kohestan, Afghanistan

**Keywords:** Klotho, FGF23, c-fos, High-protein diet, Low-calorie diet

## Abstract

**Background:**

In the current study, our specific aim was to characterize the Klotho protein and expression levels in the hippocampus and prefrontal cortex of old rats treated with different diets (high-fat, high-protein, low-calorie, high-protein and low-calorie).

**Methods:**

Rats were treated with high-fat, high-protein, low-calorie, low-calorie high-protein diets for 10 weeks and then behavioral and molecular assessments were evaluated.

**Results:**

Statistical analysis showed the percentage of open arm time was increased in the high-protein, low-calorie and low-calorie high-protein groups compared with old control (old-C) rats. The percentage of open arm entries was increased in the low-calorie and low-calorie high-protein group compared with old-C rats. The body weight and serum triglyceride were decreased in the low-calorie and low-calorie high-protein groups in comparison to control old rats. Low-calorie and low-calorie high-protein treatments statistically enhanced caspase-3 level compared with old-C rats in the hippocampus and prefrontal cortex. Treatment of old rats with high-protein, low-calorie and low-calorie high-protein could increase Klotho-α level compared with control old rats. The levels of Klotho-α, c-fos and brain-derived neurotrophic factors were decreased in the low-calorie high-protein group in Klotho inhibitor's presence compared with the low-calorie high-protein group.

**Conclusion:**

According to our findings, Klotho-α level was reduced in old rats. Low-calorie, high-protein and particularly low-calorie high-protein diets increased this protein level and consequently increased neuronal plasticity and improved memory function.

**Graphic abstract:**

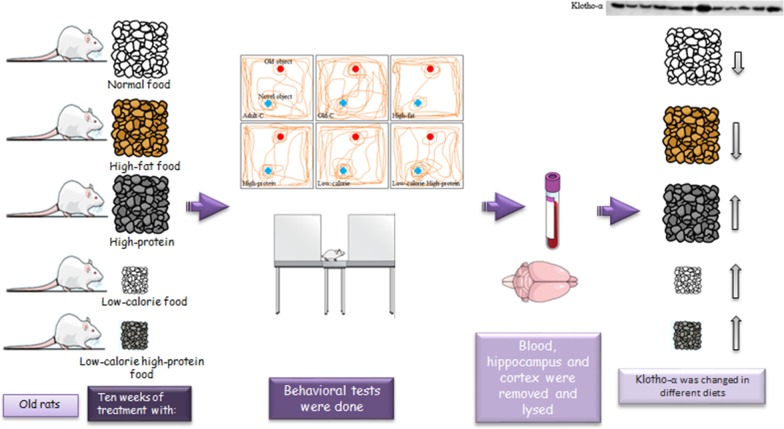

## Background

Aging is the consequence of some developments; during physiological aging, some changes happened in the body, such as decreased food and energy intake. Diet is one of the extrinsic factors, which could control our body’s metabolism [1]. Focus on dietary protocol and supplements are necessary for older people. Diet composition strongly affects health outcomes, and recently, clinical studies agreed that energy restriction with the high-protein diet could improve body metabolism, cognition and neurodegeneration [2, 3].

The anti-aging protein Klotho has been reported to negate oxidative stress and inflammation when it is overexpressed; for example, in neurodegenerative diseases, Klotho possesses anti-inflammatory properties [4]. Several studies showed a mutation in the *Klotho* gene or *Klotho* knockout mice represented the early aging signs such as short lifespan, infertility and dementia [5, 6]. Klotho is an obligate co-receptor of fibroblast growth factor (FGF) receptors (FGFRs) and Klotho/FGFRs complex act many metabolic functions in many tissues such as the kidney, heart and brain [7]. Studies have detected the downstream signaling proteins of Klotho in different pathogenic contexts; for example, it has been shown that a high-protein diet could amend fibroblast growth factor 21 (FGF21) expression in the brain, and subsequently controls dietary intake and initiates *Klotho* gene expression in the neurons, and induces neuroprotective effect against brain injury [8, 9]. Fibroblast growth factor 23 (FGF23) is a known co-receptor of Klotho in the kidney, and recently, it has been demonstrated that FGF23/Klotho complex could enhance synaptic density in the hippocampal neurons [10].

Based on different dietary protocols and unclear mechanism of Klotho in different diets, our specific aim was to characterize the role of Klotho and FGF23 proteins in the hippocampus and prefrontal cortex of old rats treated with different diets (high-fat, high-protein, low-calorie, low-calorie high-protein). We assessed behavioral tests and some neuroprotective factors such as TGFβ, c-fos and brain-derived neurotrophic factor (BDNF). Then, we inhibited the Klotho protein in the brain via the administration of d-saccharic acid 1,4-lactone and measured c-fos, BDNF and klotho protein level to found downstream proteins of Klotho.

## Methods

### Animals

Tehran University of Medical Sciences (Tehran, Iran) provided the study with 6-month and (270–300 g) and 20-month-old male adult Wistar rats (320–350 g). They were familiarized with their room in Plexiglas cages and kept under controlled light/dark cycle (12/12 h with the light beginning at 7:00 a.m.), temperature (22 ± 2 °C), food and water ad libitum. Following the international guidelines for animal experiments, the Ethics Committee of Tehran University of Medical Sciences, Tehran, Iran, approved all experimental procedures (IR.TUMS.MEDICINE.REC.1398.195).

### Experimental design

This study is designed into two experimental designs. In the first experimental design, 20-month-old and 6-month-old male rats were randomly put in six groups. All rats in each group were housed individually (n = 7): (1) adult control (adult-C), adult rats were treated with control diet for 10 weeks, (2) old control (old-C), old rats were treated with control diet for 10 weeks, (3) high-fat diet, old rats were treated with high fat food for 10 weeks, (4) high-protein diet, old rats were treated with high-protein food for 10 weeks, (5) low-calorie diet, old rats were treated with low amount of control diet for 10 weeks, (6) low-calorie high-protein diet, old rats were treated with low amount of high-protein food for 10 weeks. In the second experimental design, 20-month-old male rats were randomly put in four groups. (1) old control (old-C), old rats were treated with control diet for 10 weeks, (2) low-calorie high-protein diet, old rats were treated with low amount of high-protein food for 10 weeks, (3) old-C and d-saccharic acid 1,4-lacton diet, old rats were treated with low amount of high-protein food for 10 weeks and received d-saccharic acid 1,4-lactone twice per week for 10 weeks, (4) low-calorie diet and d-saccharic acid 1,4-lacton diet, old rats were treated with low amount of high-protein food for 10 weeks and received d-saccharic acid 1,4-lactone twice per week for 10 weeks. The first and second experimental design groups are the same second and sixth groups in the first experimental design.

### Dietary protocol

5 g/100BW of control diet was given to adult and old control rats each day for 10 weeks. In a high-protein diet, rats were given 5 g/100BW of high-protein food each day for 10 weeks [11]. In a high-fat diet, rats were given 5 g/100BW (210 kcal (kg d)) of high fat food each day for 10 weeks [12]. In a low-calorie diet, rats were given 3.5 g/100BW (140 kcal (kg d)) of control diet each day for 10 weeks. A decreased (30%) calorie diet was applied with no uneven reduction of any dietary component in low-calorie diet [13]. In a low-calorie high-protein diet, rats were given 3.7 g/100BW (140 kcal (kg d)) of high-protein food each day for 10 weeks. Table 1 shows each diet’s ingredients. The formulation of the diet was done based on the nutrient necessities of adult rats according to the guidelines of the American Institute of Nutrition (AIN-93 M). Behparvar Company (Tehran, Iran) produced normal, high-protein and high-fat diets. Table 1 shows each diet composition. The rats were given d-saccharic acid 1,4-lactone, a Klotho inhibitor orally two times per week for 10 weeks (80 mg/kg Body weight) [14].

**Table 1:** Macronutrient composition of the normal, high protein and high-fat diets which were fed to rats. Raw protein consists of casein and whey, raw fat comprises combinations of extra-virgin olive oil and butter and crude fiber consists of yellow shelled corn

Nutritional composition (g/100 g DM)	Normal	High-protein	High-fat
Raw protein	8.2	55.4	8.2
Raw fats	5.3	5.3	35.2
Crude fiber	47	4	47
Calcium	1	1	1
NaCl	5	5	5
Humidity	10	10	10
Methionine–cysteine	1.5	1	1.5
Sucrose	10	0	10
Lysine	5	11	5
vitamin mix AIN-93M	1	1	1
mineral mix AIN-93M	3.5	3.5	3.5
Soybean oil	7	10	7
Tallow	0	0	15

### Behavioral tests

#### Elevated plus maze (EPM) test

The wooden apparatus having two open and two closed arms (each 50 × 10 cm) with 40 cm high sidewalls, was placed 50 cm above the floor [15]. Following 10 weeks of treatment, rat was put in the center of raised plus maze with its head facing an open arm and left undisturbed for 5 min. All behavioral tests were done from 10:00 a.m. to 14:00 p.m. Anxiety was measured based on the total time spent in the open arms [OAT% (the ratio of times spent in the open arms to total times spent in any arms × 100)] and the number of entries into open arms [OAE% (the ratio of entries into open arms to the total entries × 100)] [16, 17].

#### Open field test

The open field opaque acrylic box (50 × 50 × 40 cm) was put in an isolated room without any objects or clues. The box floor was separated into 10 numbered squares, roughly 16 × 16 cm^2^ each for central or parietal localization. Following 10 weeks of treatment, the locomotion of each rat in the box for 5 min was tested and filmed by a camera fixed on the ceiling of the room (Maze router, Tabriz, Iran). Both maze router analysis did the scoring, and manually that was expressed as “Moving distance (meters)” and “Time spent in the central square (s)” [16, 17].

#### Novel object recognition test (NOR)

NOR was done in a square open field (50 × 50 × 40 cm). An investigator blinded to the treatment groups performed the test within 3 days. The habituation of the rats to the empty arena for 20 min was done 68 days after treatment. All behavioral tests were done from 10:00 a.m. to 14:00 p.m. On day 69, rats were let to go back to the arena with two identical objects for 6 min. On day 70, one of two familiar objects was substituted with a new one with different shapes, colors, and materials. Rats wandered in the arena for 6 min, and total time spent for each object was logged. Exploring an object was set as nosing and sniffing at it at a distance less than 2 cm. The objects and field were cleaned with 75% ethanol between each trial. On the last day, the used object was substituted, and the rats were monitored to find the possible position preference. Data was shown as the percentage of recognition index (RI) determined as follows: [time spent in exploring the novel object /time spent in exploring the two objects] × 100 [18].

### Body weight, blood glucose and triglyceride level

A digital balance (Farooq chemical, Tehran, Iran) was used for assessing body weight on days 1 and 70. The blood samples were gathered via the tail of animals. On day 1 and 10 weeks following treatment, the blood glucose monitoring kit (Glucocard01, Japan) was applied for detecting the blood glucose level. Afterward, the blood samples underwent centrifuging at 2500 rpm for 5 min for gathering the supernatants for analysis. The triglyceride detection kit (Mancompany, Tehran, Iran) was applied for assessing triglyceride levels.

### Western blot analysis

The hippocampi and prefrontal cortex were dissected and flash-frozen in liquid nitrogen and stored at − 80 °C. Before Western blot analysis, all samples’ homogenization was done in the suitable lysis buffer, [19] and total protein extract was prepared by centrifuging in 15,000 rpm for 5 min. Bradford’s method [20] was applied for measuring the protein concentration in the supernatants. After loading standardized lysates equivalent to 60 µg of protein on SDS-12.5% poly acrylamide gel electrophoresis and transferring to PVDF membrane (Chemicon Millipore Co. Temecula, USA), blots were blocked in 2% Electrochemiluminescence (ECL) advanced kit blocking reagent (Amersham Bioscience Co. Piscataway, USA) and probed with Klotho-α, FGF23, BDNF and c-fos (1/1000, MyBioSource Inc. CA, USA) antibodies overnight. Then, the membranes' incubation was done with rabbit IgG-horseradish peroxidase-conjugated secondary antibody (1/3000, Cell Signaling Technology Co. New York, USA) that was directly noticeable by chemiluminescence kit reagent (Amersham Bioscience Co. Piscataway, USA). For detecting β-actin as an internal control, stripping buffer (pH 6.7) was used to strip the blots and then probing was done with anti β-actin antibody (1/1000, Cell Signaling Technology Co. New York, USA) [19].

### Caspase-3 and TGF-β1 level

Caspase‐3 and TGF-β1 Elisa assay kits (Abcam, Cambridge, MA, USA) were applied based on the manufacturer's protocol to determine caspase‐3 level. A specific lysis buffer was used to lyse the hippocampi and prefrontal cortex tissues, and lysate underwent centrifuging at 15,000 rpm for 5 min at 4 °C, and the Bradford dye method [21] was applied for determining the protein concentration in the supernatants. Caspase-3 and TGF-β1 activity was assessed by measuring the absorbance at 405 nm.

### Real time PCR

Step One Plus Real-Time PCR System (Applied Biosystems) was applied for real time PCR reaction for hippocampi and prefrontal cortex of Klotho α gene. Mixing of 2 μl of cDNA, 2 μl of primer and SYBR Green Master Mix (Takara, Japan) was done following manufacture protocol in total volume of 20 μl. The annealing temperature for all primer pairs was set at 58 °C. The PCR product specificity was verified by confirming a single peak in the melting curve and visualizing 2% agarose gel with ethidium bromide in gel documentation. The cycle at which the sample fluorescence faced a predetermined threshold (Ct) considerably beyond the background was used for determining the quantity of *cfos* and *bdnf* gene for each sample. Samples were tested in duplicate and the mean was used for further analysis. The housekeeping gene (β-actin) was applied for normalizing all the data of sample and control groups.

### Sections preparation and immunofluorescence

PBS was perfused through the ascending aorta with PBS, followed by fresh 4% paraformaldehyde in PBS (pH 7.4) (n = 4). The brains were removed quickly and put in 4% paraformaldehyde in PBS for 24 h. After embedding the tissues with paraffin, they were sectioned at a thickness of 5 μm. Then, incubation was done with normal goat serum (1:100, Cell Signaling Technology Co. New York, USA); next, Klotho-α-specific antibody (1:100 dilution) covered the sections overnight at 4 °C followed by fluorescent secondary antibody Alexa Fluor 488-conjugated anti-rabbit IgG (1:500, Molecular Probes, Invitrogen, CA). A fluorescence microscope (Olympus IX71, Japan) was used for capturing the figures.

### Statistical analysis

Obtained data were expressed as mean ± S.E.M for behavioral and mean ± S.D for molecular data. Immunofluorescence staining and Western blotting blots were analyzed by quantification of densitometry data using ImageJ software (National Institutes of Health, Bethesda, MD, USA). Kolmogorov–Smirnov test was performed to find normal assumption of data. Behavioral and Western blot results were analyzed statistically by One-way ANOVA followed by Tukey’s multiple comparison; body weight, blood glucose and serum triglyceride were analyzed by repeated measure test. Correlations of assessed proteins were measured by Pearson coefficients correlation and Principal component analysis. All mentioned parameters were analyzed by SPSS software (Version 21). Data of gene expression experiments were analyzed by Relative Expression Software Tool (REST)-XL (Version 2) [22], which is used to determine significant differences in relative expression levels between sample and control groups. P value less than 0.05 is considered statistically significant.

## Results

### High-fat diet has negative and low-calorie high-protein diet has positive effect on anxiety-like behavior, locomotion, and working memory in the old rats

**Fig. 1:**
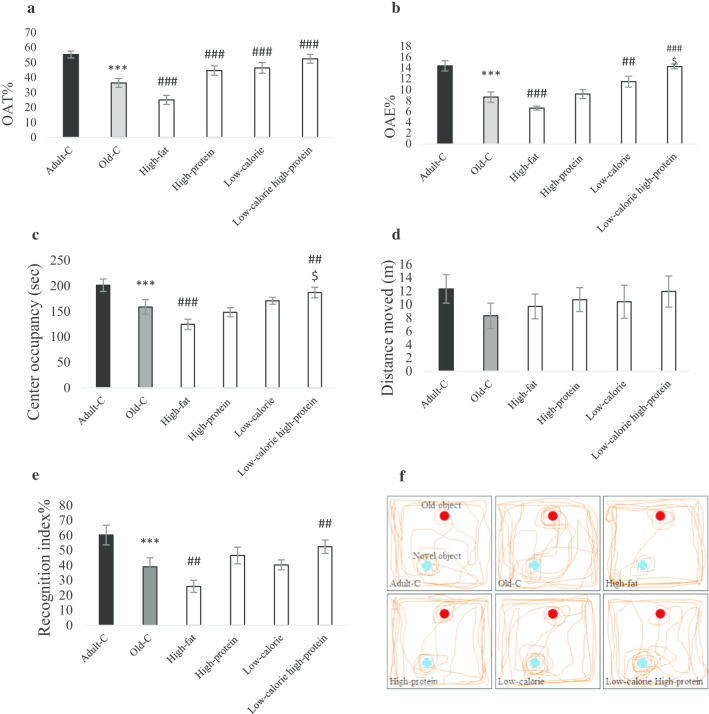
Behavioral assessments. Rats treated with high-fat, high-protein, low-calorie, low-calorie high-protein diets for 10 weeks. Control adult and old rats treated with rodent standard pellet. After 10 weeks, behavioral tests were performed (n = 7). Anxiety-like behavior was assessed by elevated plus maze test (EPM) for 5 min. Percentage of open arm time (**a**) and percentage of open arm entires (**b**) in the EPM were shown. Center occupancy (**d**) and locomotion (**c**) were assessed by open field test for 5 min (n = 7). Percentage of recognition index (**e**) was measured by novel object recognition test (n = 7). A represented tracks showing 2 min path in the NOR (**f**). Data were presented as Mean ± S.E.M. ***P < 0.001 ver. Adult-C, ##*P* < 0.01, ###*P* < 0.001 ver. Old-C. $*P* < 0.05 ver. High-protein. *Adult-C* adult control rats, *Old-C* old control rats

Figure 1a–c demonstrates the anxiety-like behavior measured by EPM and open field tests (n = 7). Percentages of OAT, OAE, and time of center occupancy were decreased in old-C rats compared with adult-C (*P* < 0.001). Percentage of OAT was increased in the high-protein, low-calorie and low-calorie high-protein groups compared with old-C rats. Percentage of OAE was increased in the low-calorie (*P* = 0.004) and low-calorie high-protein (*P* < 0.001) group compared with old-C rats. Percentage of OAE was increased in the low-calorie high-protein groups compared with high-protein group (*P* < 0.05). Time of center occupancy was increased in the low-calorie high-protein group compared with control old rats (Fig. 1d, *P* = 0.005) and high-protein group (*P* < 0.05). Figure 1e, f show that the old rats’ recognition index was decreased compared to adult rats (n = 7, *P* < 0.001). Statistical analysis showed that high-fat diet decreased the recognition index in the old rats compared with control diet group. Treatment of old rats with low-calorie high-protein diet could enhance the recognition index compared with control diet group (*P* = 0.003).

### Body weight, blood glucose and serum triglyceride are changed in low-calorie diet and high-protein of old rats

Table 2 shows the body weight, blood glucose, and serum triglyceride level in the experimental groups before and after 10 weeks of treatment (n = 7). Before treatment, body weight, blood glucose and serum triglyceride of old-C rats were lower than adult-C rats (*P* < 0.05). High-protein treatment increased body weight (*P* = 0.005) and decreased serum triglyceride (*P* = 0.002) in comparison to control old rats. Data analysis showed the body weight and serum triglyceride were decreased in the low-calorie and low-calorie high-protein groups in comparison to control old rats. The blood glucose was decreased in the low-calorie and low-calorie high-protein compared with control old rats (*P* = 0.03).

**Table 2:** Body weight, blood glucose and serum triglyceride level in experimental groups. Rats treated with high-fat, high-protein, low-calorie, low-calorie high-protein diets for 10 weeks. Control adult and old rats treated with rodent standard pellet. After behavioral tests, rats were euthanized and blood were collected (n = 6)

Groups	Body weight (g)		Blood glucose (mg dl)		Serum triglyceride (mg dl)	
	Before treatment	After treatment	Before treatment	After treatment	Before treatment	After treatment
Adult-C	302 ± 22	343 ± 31	85 ± 2	87 ± 3	81 ± 3	84 ± 2
Old-C	367 ± 11^$^	396 ± 22	98 ± 4^$^	105 ± 4	95 ± 2^$^	96 ± 2
High-fat	350 ± 15	379 ± 25	95 ± 4	110 ± 5	93 ± 5	119 ± 5
High-protein	333 ± 27	405 ± 19**	94 ± 2	99 ± 5	96 ± 2	79 ± 3**
Low-calorie	359 ± 16	315 ± 18*	98 ± 3	90 ± 6*	94 ± 6	70 ± 3**
Low-calorie high-protein	337 ± 21	307 ± 16*	99 ± 3	90 ± 5*	91 ± 5	63 ± 4***

^$^*P* < 0.05 versus adult-c group, *P < 0.05, **P < 0.01, ***P < 0.001 versus before treatment in the same group

### Different diets change caspase-3 and TGF-β1 level in the hippocampus and prefrontal cortex

Caspase-3 and TGF-β1 levels were detected in the hippocampus and prefrontal cortex (n = 4, Table 3). Data reported that caspase-3 level was increased in the hippocampus of old-C rats compared with adult-c rats (*P* < 0.001). In addition, the TGF-β1 level was reduced in the hippocampus and prefrontal cortex of old-C rats compared with adult-c rats (*P* < 0.001). High-fat treatment increased caspase-3 and TGF-β1 level compared with old-C rats in the prefrontal cortex. Low-calorie (*P* = 0.02) and low-calorie high-protein (*P* < 0.001) treatments reduced caspase-3 level compared with old-C rats in the hippocampus and prefrontal cortex. High-protein (*P* = 0.02), low-calorie (*P* = 0.04) and low-calorie high-protein (*P* < 0.001) treatments improved TGF-β1 level compared with old-C rats in the hippocampus and prefrontal cortex. High-protein low-calorie treatments improved TGF-β1 level compared with high-protein group in the hippocampus and prefrontal cortex (*P* < 0.05).

**Table 3:** Caspase-3 activity and TGF-β1level in the hippocampus and prefrontal of experimental groups were detected by Elisa assay kits. Rats treated with high-fat, high-protein, low-calorie, low-calorie high-protein diets for 10 weeks. Control adult and old rats treated with rodent standard pellet. After behavioral tests, rats were euthanized and brains were collected (n = 3)

Groups	Caspase-3 (U.g)		TGF-β1(ng.g)	
	Hippocampus	Prefrontal cortex	Hippocampus	Prefrontal cortex
Adult-C	6.1 ± 0.2	5.0 ± 0.4	6.8 ± 0.2	5.0 ± 0.4
Old-C	6.8 ± 0.3***	5.4 ± 0.6	2.8 ± 0.3***	2.3 ± 0.3***
High-fat	7.1 ± 0.3	6.9 ± 0.2^##^	3.2 ± 0.5	3.5 ± 0.2^#^
High-protein	6.5 ± 0.4	5.5 ± 0.4	5.6 ± 0.5^##^	4.1 ± 0.7^##^
Low-calorie	6.2 ± 0.6^#^	4.8 ± 0.3^#^	7.1 ± 0.4^##^	5.5 ± 0.4^##^
Low-calorie high-protein	6.0 ± 0.3^###^	4.5 ± 0.3^###^	7.5 ± 0.8^$###^	5.9 ± 0.2^$###^

***P < 0.001 versus Adult-C. ^#^*P* < 0.05, ^##^*P* < 0.01, ^###^*P* < 0.001 versus Old-C. ^$^*P* < 0.05 versus high-protein group

### Klotho-α and FGF23 were improved by high-protein, low-calorie and low-calorie high-protein diets in the hippocampus and prefrontal cortex of old rats

**Fig. 2:**
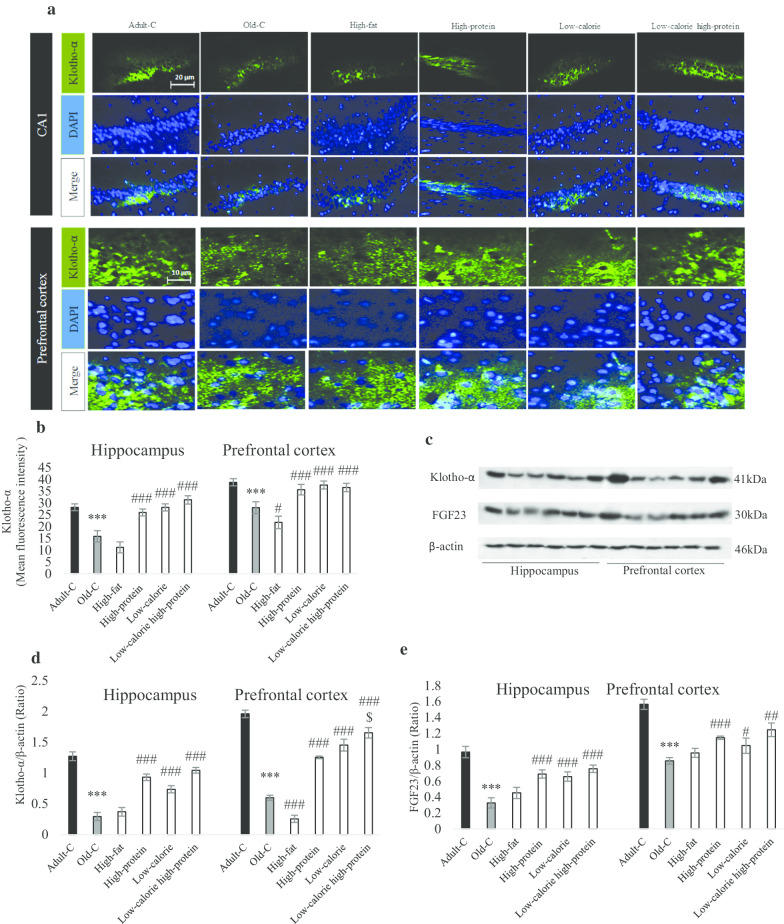
Molecular assessment of Klotho-α and FGF23 in the hippocampus and prefrontal cortex of experimental groups. Rats treated with high-fat, high-protein, low-calorie, low-calorie high-protein diets for 10 weeks. Control adult and old rats treated with rodent standard pellet. After behavioral tests, rats were euthanized and brains of three of them perfused for histological evaluation in each group (n = 3) and brains of other three rats in each groups were collected for Western blotting technique (n = 4). Immunoflorence assay **a** showed the Klotho-α distribution in the hippocampus and prefrontal cortex. Mean of fluorescence intensity showed Klotho-α positive cell in the CA1 and prefrontal cortex (**b**). A represented blot showed Klotho-α and FGF23 protein level (**c**) and the density of Klotho-α bands (**d**) and FGF23 band (**e**) were measured in the hippocampus and prefrontal cortex (n = 4 and technical repeat for each n is 3). Data were presented as Mean ± S.D. ***P < 0.001 ver. Adult-C, #*P* < 0.05, ##*P* < 0.01, ###*P* < 0.001 ver. Old-C. $*P* < 0.05 ver. High-protein. *Adult-C* adult control rats, *Old-C* old control rats

**Fig. 3:**
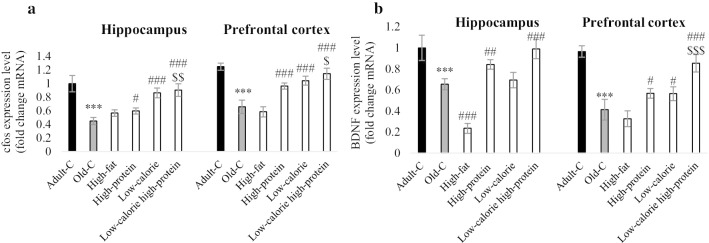
Gene expression of c-fos (**a**) and BDNF (**b**) in the hippocampus and prefrontal cortex (n = 4). Rats treated with high-fat, high-protein, low-calorie, low-calorie high-protein diets for 10 weeks. Control adult and old rats treated with rodent standard pellet. After behavioral tests, rats were euthanized and brains of three rats in each groups were collected for RT-PCR (n = 4 and technical repeat for each n is 3). Data were presented as Mean ± S.D. ***P < 0.001 ver. Adult-C, ^#^*P* < 0.05, ^##^*P* < 0.01, ^###^*P* < 0.001 ver. Old-C. ^$^*P* < 0.05, ^$$^*P* < 0.01, ^$$$^*P* < 0.001 ver. High-protein. *Adult-C* adult control rats, *Old-C* old control rats

As shown in Fig. 2a, b, the amount of Klotho-α was reduced in the old-C rats compared with adult-C group (*P* < 0.001). Mean of Florence intensity in the prefrontal cortex showed high-fat diet decreased Klotho-α in comparison to control old rats (*P* = 0.02). Treatment of old rats with high-protein, low-calorie and low-calorie high-protein could increase Klotho-α level compared with control old rats (*P* < 0.001). Figure 3c represented a blot of Klotho-α and FGF23; densitometry analysis in Fig. 2d, e reported high-protein, low-calorie and low-calorie high-protein increased Klotho-α and FGF23 levels compared with control old rats in the both hippocampus and prefrontal cortex. Also, Klotho-α level was increased in low-calorie high-protein group compared with high-protein group in the prefrontal cortex (*P* < 0.05).

### High-protein, low-calorie and low-calorie high-protein diets could improve c-fos and BDNF level in the brain of old rats

RT-PCR data was used to evaluate c-fos and BDNF gene expression in the hippocampus and prefrontal cortex of experimental groups (Fig. 3a, b). Results showed that both c-fos and BDNF expression were decreased in the old-C rats compared with adult-C rats (*P* < 0.001). Treatment of old rats with high-protein, low-calorie, and low-calorie high-protein diets improved c-fos level compared with control old rats in the hippocampus and prefrontal cortex. c-fos level was increased in low-calorie high-protein group compared with high-protein group in the both hippocampus (*P* < 0.01) and prefrontal cortex (*P* < 0.05). Level of BDNF was increased in the high-protein and low-calorie high-protein groups compared with control old rats in the hippocampus and prefrontal cortex. The high-fat diet reduced hippocampal BDNF in comparison to control old rats (*P* < 0.001). Low-calorie diet increased prefrontal cortex BDNF compared with control old rats (*P* < 0.001). BDNF level was increased in low-calorie high-protein group compared with high-protein group in the prefrontal cortex (*P* < 0.001).

### Inhibition of Klotho-α reduced neuronal plasticity in low-calorie high-protein diet received old rats

**Fig. 4:**
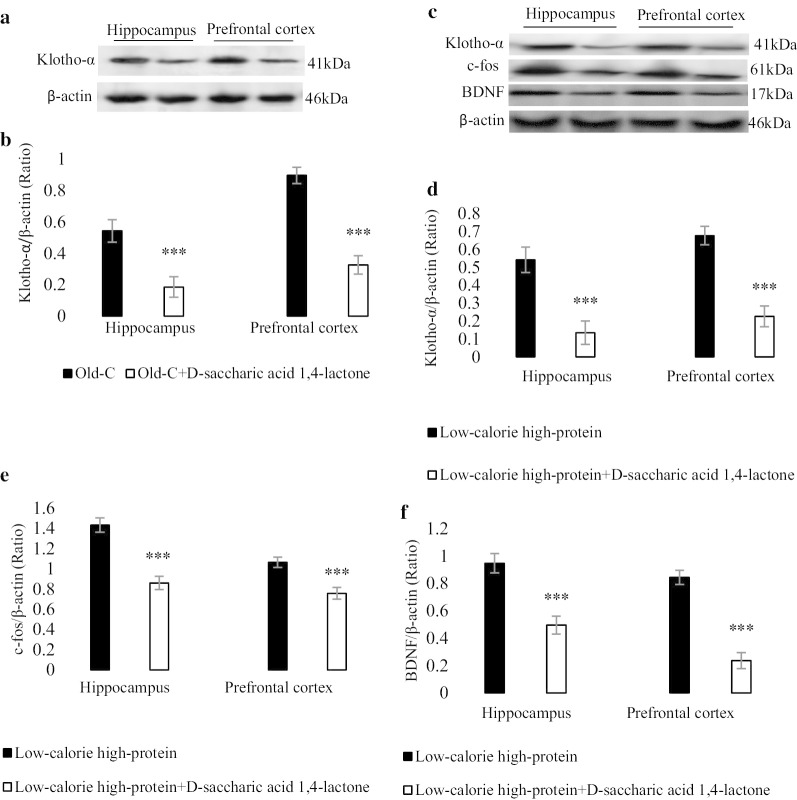
Molecular assessment of Klotho-α, c-fos and BDNF in the hippocampus and prefrontal cortex of experimental groups (n = 4). A represented blot showed Klotho-α in the old rats with standard diet for 10 weeks and old rats with Klotho-α inhibitor (d-saccharic acid 1,4-lactone, orally administrated 2 time/week for 10 weeks) (**a**) and the density of Klotho-α band (**b**) was measured. A represented blot showed Klotho-α, c-fos and BDNF in the low-calorie high-protein group (treated for 10 weeks) with and without Klotho-α inhibitor (**c**) and the density of Klotho-α (**d**), c-fos (**e**) and BDNF (**f**) bands were measured in the hippocampus and prefrontal cortex. Data were presented as Mean ± S.D. ***P < 0.001 ver. low-calorie high-protein group. *Adult-C* adult control rats, *Old-C* old control rats

Figure 4a, b show the Klotho inhibitor (d-saccharic acid 1,4-lactone), inhibited the Klotho-α in the hippocampus and prefrontal cortex of old rats. Figure 4c shows a represented blot of Klotho-α, c-fos and BDNF in low-calorie high-protein group in the presence of Klotho inhibitor. The levels of Klotho-α, c-fos and BDNF were decreased in the low-calorie high-protein group in the presence of Klotho inhibitor compared with low-calorie high-protein group (*P* < 0.001).

### Correlation analysis of Klotho protein and other measured genes and proteins

Table 4 shows that Pearson coefficients correlation showed a correlation between Klotho protein level in the brain and all assessed proteins or genes (FGF23, TGF-β, caspase-3, c-fos and BDNF). Data analysis indicated there is significant correlation between Klotho and all mentioned factors.

**Table 4:** Correlation between Klotho protein and FGF23 protein, TGF-β protein, caspase-3 protein, c-fos and BDNF gene expression (n = 3 and technical replication is 3)

Correlation measured factors (Pearson coefficients correlation)				
FGF23	TGF-β	Caspase-3	c-fos gene expression	BDNF gene expression
Klotho				
Hippocampus				
r = 0.81	r = 0.65	r = 0.83	r = 0.81	r = 0.64
*P* = 0.000	*P* = 0.003	*P* = 0.001	*P* = 0.000	*P* = 0.000
Prefrontal cortex				
r = 0.63	r = 0.64	r = 0.80	r = 0.80	r = 0.66
*P* = 0.000	*P* = 0.001	*P* = 0.001	*P* = 0.000	*P* = 0.000

*P* value less than 0.05 considered significant

## Discussion

It has been well accepted that consumption of low-quality diets are associated with higher frailty in humans and animals [23]; moreover, it is believed that some dietary patterns such as high-protein and calorie-restricted diets can suppress neurodegeneration with aging [24, 25]. However, little is known about the influence of dietary patterns on the molecular process of aging. Herein, we showed the differences between adult and old rats in terms of movement and anxiety disorders, and focused on three types of diets (high-fat, low-calorie, high-protein and low-calorie high-protein). We found deleterious effects of high-fat diet and beneficial influence of low-calorie, high-protein and low-calorie high-protein diets on the aging process. Low-calorie and low-calorie high-protein diets could reduce body weight, blood glucose and serum triglyceride level. On the other hand, high-protein treatment only decreases serum triglyceride levels. Arbo et al. [26], confirmed our data and demonstrated old rats have metabolic syndrome, and a high-calorie diet increases the serum triglyceride and body weight, showing the importance of a low-calorie diet against aging. Besides, studies proved that calorie-restriction decreases and high-fat diet increases serum triglyceride and blood glucose [27, 28]. A randomized trial showed a high-protein diet could improve body weight, blood glucose and serum triglyceride [29]; but our observation revealed a high-protein diet only could decrease serum triglyceride. Many studies indicated different protein types influence energy intake and body weight in rats receiving a high-protein diet [30, 31], and high-protein diet with either moderate or low carbohydrate has a favorable impact on metabolic health [32], which is in parallel with our finding that low-calorie high-protein diet has more effect rather than high-protein diet in metabolic factors. Also, it has been reported high-protein diet impaired liver function [33]; however, in our study, not only all three factors-high-protein, low calorie and low-calorie high-protein diets- have no degenerative role on liver function, but also, klotho-α level increased in liver, which showed the protective role of these three diets on body metabolism (under publishing data).

Results of the present study confirmed old rats have less anxiolytic, locomotive and memory function in comparison to young rats, which highlighting the impairment of cognitive function in old rats, [34] and high-fat diet worsens anxiety-like behavior and working memory in old rats, but, in contrast, high-protein, low-calorie and low-calorie high-protein diets improved anxiety. Low-calorie high-protein diet could increase the recognition index as a representative of working memory. Clinical and experimental studies found that a high-fat diet impairs cognitive function and increases anxiety in rodents and healthy human [35–38]. In which these findings are in agreement with our data that the high-fat diet induces anxiety-like behavior in aged rats. On the other hand, calorie restriction is one of the most focused dietary patterns to improve cognition in the elderly [39, 40] and current results established low-calorie high-protein diet had more influence on memory compared with low-calorie regular food.

Anti-aging protein Klotho and its co-receptor, FGF23 were boosted in the hippocampus and prefrontal cortex when rats were treated with high-protein, low-calorie and low-calorie high-protein diets confirming the role of the dietary protocol against anti-aging proteins [41]. The role of different diets on aging has been established and the Klotho signaling has been represented as an essential factor against aging [42]. Rios et al. [43] indicated that the high-fat diet reduced Klotho and FGF23 in the kidney. A clinical study showed the high-protein diet could improve Klotho and FGF21 serum level [44]; also, the low carbohydrate high-protein diet could induce FGF signaling and neuroprotection [45]. Data displayed low-calorie high-protein diet has a more influential role in klotho-α expression in the prefrontal cortex; these findings propose low-calorie high-protein diet is one of the promising candidates against neurodegeneration.

Also, we established Klotho-α level was increased in the low-calorie, high-protein and low-calorie high-protein diets in the serum, which confirmed Klotho-α circulate in the whole body and act their anti-aging function in many tissues such as the liver [46]. To find the precise role of diets on brain function, we evaluated the caspase-3 and some factors involved in neuronal plasticity in the hippocampus and prefrontal cortex. Data revealed caspase-3 level was increased during aging in the hippocampus, confirming the hippocampus’s vulnerability rather than the prefrontal cortex [47]; moreover, high-fat diet increased caspase-3 level and low-calorie and low-calorie high-protein diets inhibited the caspase-3 activity in both hippocampus and prefrontal cortex. Neuronal plasticity factors such as TGF-β1, c-fos and BDNF were decreased during aging and high-fat diet intensifies this reduction in the brain; in contrast, high-protein, low-calorie and low-calorie high-protein diets could enhance these factors suggesting the role of these diets against neuronal degeneration and cognition [48, 49]. Furthermore, data analysis indicated low-calorie high-protein diet has a more beneficial role in cognitive-related factors than a high-protein diet, confirmed by the in vivo and clinical studies [50, 51].

According to our data, low-calorie and low-calorie high-protein diets are the most protective diet, so we chose low-calorie high-protein diet for the second step of the experimental procedure, and Klotho inhibitor, d-saccharic acid 1,4-lactone has been used. Inhibition of Klotho decreased c-fos and BDNF level in the low-calorie high-protein received rats, which established the vital role of different diets against neurodegeneration. FGF/Klotho has a potential effect on c-fos in the kidney and liver [52, 53], and these results showed that Klotho/c-fos signaling is presented in the brain. Inhibition of Klotho reduces BDNF and c-fos neuronal level, which shows BDNF and c-fos are downstream signal transduction for Klotho and might enhance working memory in the old rats. Previous studies well establish the role of BDNF in the Klotho signaling, and it has been shown that mutation of Klotho decreases BDNF level and also inhibition of BDNF could impair cognitive dysfunction in the Klotho mutant mice [54]. It is noteworthy that the d-saccharic acid 1,4-lactone inhibits both Klotho-α and -β, so the future study is suggested to be done to explore the effect Klotho-α inhibition on diets by using a specific inhibitor of Klotho-α or *Klotho-α* gene knockout.

## Conclusion

Data found diets such as high-protein, low-calorie, low-calorie high-protein diets increased Klotho/FGF23 signaling in the brain, and consequently, Klotho could enhance neuronal plasticity factors and enhance memory and anxiety. The result of the current study can be used by clinical practice and helps understand the anxiety-like disorder’s mechanisms and treatments. Also, many shreds of evidence support that the ketogenic diet, which contains low-carbohydrate high-protein or calorie-restricted diets containing low-carbohydrate, could improve cognition in healthy or aged humans. On the other hand, the study was conducted in male rats, and it might not be easy to extrapolate the results to female rats. Taken together, data confirmed that low-calorie and low-calorie high-protein diets could potentially reduce aging and cognitive deficits by elevation of Klotho (Graphical abstract).

## Data Availability

The data sets used and/or analyzed during the current study are available from the corresponding author on reasonable request.

## Abbreviations

OAT: Open arm time
OAE: Open arm entries
FGF23: Fibroblast growth factor-23
BDNF: Brain-derived neurotrophic factor
EPM: Elevated plus maze
NOR: Novel object recognition
VDF: Polyvinylidene fluoride;
CL: Electrochemiluminescence
TGF-β: Transforming growth factor beta
T-PCR: Real time polymerase chain reaction
REST: Relative Expression Software Tool
BS: Phosphate-buffered saline
NOVA: Analysis of variance
S.D: Standard deviation

## References

[1] Mehta HH, Xiao JL, Ramirez R, Miller B, Kim SJ (2019). Metabolomic profile of diet-induced obesity mice in response to humanin and small humanin-like peptide 2 treatment. Metabolomics.

[2] Paoli A, Bianco A, Damiani E, Bosco G (2014). Ketogenic diet in neuromuscular and neurodegenerative diseases. Biomed Res Int.

[3] Mero AA, Huovinen H, Matintupa O, Hulmi JJ, Puurtinen R (2010). Moderate energy restriction with high protein diet results in healthier outcome in women. J Int Soc Sports Nutr.

[4] Kurosu H, Yamamoto M, Clark JD, Pastor JV, Nandi A (2005). Suppression of aging in mice by the hormone Klotho. Science.

[5] Kuroo M, Matsumura Y, Aizawa H, Kawaguchi H, Suga T (1997). Mutation of the mouse klotho gene leads to a syndrome resembling ageing. Nature.

[6] Flicker L, Morar B, Hankey G, Yeap B, Golledge J (2017). Longevity klotho gene polymorphism and the risk of dementia in older men. Austr J Ageing.

[7] Richter B, Faul C (2018). FGF23 actions on target tissues-with and without Klotho. Front Endocrinol (Lausanne).

[8] Larson KR, Chaffin ATB, Goodson ML, Fang YB, Ryan KK (2019). Fibroblast growth factor-21 controls dietary protein intake in male mice. Endocrinology.

[9] Ye LX, Wang X, Cai CC, Zeng SS, Bai JJ (2019). FGF21 promotes functional recovery after hypoxic-ischemic brain injury in neonatal rats by activating the PI3K/Akt signaling pathway via FGFR1/beta-klotho. Exp Neurol.

[10] Hensel N, Schon A, Konen T, Lubben V, Forthmann B (2016). Fibroblast growth factor 23 signaling in hippocampal cells: impact on neuronal morphology and synaptic density. J Neurochem.

[11] Aparicio VA, Nebot E, Garcia-del Mora R, Machado-Vilchez M, Porres JM (2013). High-protein diets and renal status in rats. Nutr Hosp.

[12] Sahin K, Tuzcu M, Orhan C, Sahin N, Kucuk O (2013). Anti-diabetic activity of chromium picolinate and biotin in rats with type 2 diabetes induced by high-fat diet and streptozotocin. Br J Nutr.

[13] Nangaku M, Izuhara Y, Usuda N, Inagi R, Shibata T (2005). In a type 2 diabetic nephropathy rat model, the improvement of obesity by a low calorie diet reduces oxidative/carbonyl stress and prevents diabetic nephropathy. Nephrol Dial Transplant.

[14] Bhattacharya S, Manna P, Gachhui R, Sil PC (2013). d-Saccharic acid 1,4-lactone protects diabetic rat kidney by ameliorating hyperglycemia-mediated oxidative stress and renal inflammatory cytokines via NF-kappa B and PKC signaling. Toxicol Appl Pharmacol.

[15] Menard J, Treit D (1998). The septum and the hippocampus differentially mediate anxiolytic effects of R(+)-8-OH-DPAT. Behav Pharmacol.

[16] Pietrelli A, Lopez-Costa J, Goni R, Brusco A, Basso N (2012). Aerobic exercise prevents age-dependent cognitive decline and reduces anxiety-related behaviors in middle-aged and old rats. Neuroscience.

[17] Gupta YK, Sinha K, Chaudhary G (2002). Transient focal ischemia induces motor deficit but does not impair the cognitive function in middle cerebral artery occlusion model of stroke in rats. J Neurol Sci.

[18] Martin-Moreno AM, Brera B, Spuch C, Carro E, Garcia-Garcia L (2012). Prolonged oral cannabinoid administration prevents neuroinflammation, lowers beta-amyloid levels and improves cognitive performance in Tg APP 2576 mice. J Neuroinflamm.

[19] Niimura M, Takagi N, Takagi K, Mizutani R, Ishihara N (2006). Prevention of apoptosis-inducing factor translocation is a possible mechanism for protective effects of hepatocyte growth factor against neuronal cell death in the hippocampus after transient forebrain ischemia. J Cereb Blood Flow Metab.

[20] Bradford MM (1976). A rapid and sensitive method for the quantitation of microgram quantities of protein utilizing the principle of protein-dye binding. Anal Biochem.

[21] Lam TG, Jeong YS, Kim SA, Ahn SG (2018). New metformin derivative HL156A prevents oral cancer progression by inhibiting the insulin-like growth factor/AKT/mammalian target of rapamycin pathways. Cancer Sci.

[22] Pfaffl MW, Horgan GW, Dempfle L (2002). Relative expression software tool (REST) for group-wise comparison and statistical analysis of relative expression results in real-time PCR. Nucleic Acids Res.

[23] Fried LP, Tangen CM, Walston J, Newman AB, Hirsch C (2001). Frailty in older adults: evidence for a phenotype. J Gerontol Ser A Biol Sci Med Sci.

[24] Mobbs CV, Mastaitis J, Yen K, Schwartz J, Mohan V (2007). Low-carbohydrate diets cause obesity, low-carbohydrate diets reverse obesity: a metabolic mechanism resolving the paradox. Appetite.

[25] Davidenko O, Darcel N, Fromentin G, Tome D (2013). Control of protein and energy intake—brain mechanisms. Eur J Clin Nutr.

[26] Arbo BD, Niches G, Zanini P, Bassuino DM, Driemeier D (2018). Aging affects the response of female rats to a hypercaloric diet. Exp Gerontol.

[27] Sun QQ, Nie SS, Wang LX, Yang F, Meng ZM (2016). Factors that affect pancreatic islet cell autophagy in adult rats: evaluation of a calorie-restricted diet and a high-fat diet. PLoS ONE.

[28] Margolis LM, Rivas DA, Ezzyat Y, Gaffney-Stomberg E, Young AJ (2016). Calorie restricted high protein diets downregulate lipogenesis and lower intrahepatic triglyceride concentrations in male rats. Nutrients.

[29] Bowen J, Brindal E, James-Martin G, Noakes M (2018). Randomized trial of a high protein, partial meal replacement program with or without alternate day fasting: similar effects on weight loss, retention status, nutritional, metabolic, and behavioral outcomes. Nutrients.

[30] Karalazos V, Bendiksen EA, Dick JR, Tocher DR, Bell JG (2011). Influence of the dietary protein:lipid ratio and fish oil substitution on fatty acid composition and metabolism of Atlantic salmon (*Salmo salar*) reared at high water temperatures. Br J Nutr.

[31] Pichon L, Potier M, Tome D, Mikogami T, Laplaize B (2008). High-protein diets containing different milk protein fractions differently influence energy intake and adiposity in the rat. Br J Nutr.

[32] Lobley GE, Bremner DM, Holtrop G, Johnstone AM, Maloney C (2007). Impact of high-protein diets with either moderate or low carbohydrate on weight loss, body composition, blood pressure and glucose tolerance in rats. Br J Nutr.

[33] Remesy C, Demigne C (1982). Impaired lactate utilization in livers of rats fed high protein-diets. J Nutr.

[34] Balietti M, Pugliese A, Fabbietti P, Di Rosa M, Conti F (2019). Aged rats with different performances at environmental enrichment onset display different modulation of habituation and aversive memory. Neurobiol Learn Mem.

[35] Magnusson KR, Hauck L, Jeffrey BM, Elias V, Humphrey A (2015). Relationships between diet-related changes in the gut microbiome and cognitive flexibility. Neuroscience.

[36] Tsai SF, Wu HT, Chen PC, Chen YW, Yu MG (2018). High-fat diet suppresses the astrocytic process arborization and downregulates the glial glutamate transporters in the hippocampus of mice. Brain Res.

[37] Holloway CJ, Cochlin LE, Emmanuel Y, Murray A, Codreanu I (2011). A high-fat diet impairs cardiac high-energy phosphate metabolism and cognitive function in healthy human subjects. Am J Clin Nutr.

[38] Almeida-Suhett CP, Scott JM, Graham A, Chen YF, Deuster PA (2019). Control diet in a high-fat diet study in mice: Regular chow and purified low-fat diet have similar effects on phenotypic, metabolic, and behavioral outcomes. Nutr Neurosci.

[39] Kuhla A, Lange S, Holzmann C, Maass F, Petersen J (2013). Lifelong caloric restriction increases working memory in mice. PLoS ONE.

[40] Diniz DB, de Oliveira SL, Melo LL, Amaya-Farfan J (2009). Comparing the impact of chronic energy restriction and vitamin E supplementation on the behavior of adult rats. Arch Latinoam Nutr.

[41] Fujitsuka N, Asakawa A, Morinaga A, Amitani MS, Amitani H (2016). Increased ghrelin signaling prolongs survival in mouse models of human aging through activation of sirtuin1. Mol Psychiatry.

[42] Tsujikawa H, Kurotaki Y, Fujimori T, Fukuda K, Nabeshima YI (2003). Klotho, a gene related to a syndrome resembling human premature aging, functions in a negative regulatory circuit of vitamin D endocrine system. Mol Endocrinol.

[43] Rios R, Pineda C, Lopez I, Munoz-Castaneda J, Rodriguez M (2018). Phosphorus restriction does not prevent the increase in fibroblast growth factor 23 elicited by high fat diet. PLoS ONE.

[44] Markova M, Pivovarova O, Hornemann S, Sucher S, Frahnow T (2017). Isocaloric diets high in animal or plant protein reduce liver fat and inflammation in individuals with type 2 diabetes. Gastroenterology.

[45] Badman MK, Pissios P, Kennedy AR, Koukos G, Flier JS (2007). Hepatic fibroblast growth factor 21 is regulated by PPARalpha and is a key mediator of hepatic lipid metabolism in ketotic states. Cell Metab.

[46] Rahimi A, Shafie A, Nabavizadeh F, Ashabi G (2020). Comparison of effects of high-fat, high-protein and low-calorie diets on Klotho gene expression and TGF-β level in serum and liver of old male Wistar rats. Iran J Nutr Sci Food Technol..

[47] McEwen BS, Nasca C, Gray JD (2016). Stress effects on neuronal structure: hippocampus, amygdala, and prefrontal cortex. Neuropsychopharmacology.

[48] Janssen CIF, Jansen D, Mutsaers MPC, Dederen PJWC, Geenen B (2016). The effect of a high-fat diet on brain plasticity, inflammation and cognition in female ApoE4-knockin and ApoE-knockout mice. PLoS ONE.

[49] Holt RL, Mikati MA (2011). Care for child development: basic science rationale and effects of interventions. Pediatr Neurol.

[50] Oarada M, Tsuzuki T, Nikawa T, Kohno S, Hirasaka K (2012). Refeeding with a high-protein diet after a 48 h fast causes acute hepatocellular injury in mice. Br J Nutr.

[51] Buhl SF, Beck AM, Christensen B, Caserotti P (2020). Effects of high-protein diet combined with exercise to counteract frailty in pre-frail and frail community-dwelling older adults: study protocol for a three-arm randomized controlled trial. Trials.

[52] Du E, Xiao L, Hurley MM (2017). FGF23 neutralizing antibody ameliorates hypophosphatemia and impaired FGF receptor signaling in kidneys of HMWFGF2 transgenic mice. J Cell Physiol.

[53] Lin BC, Wang M, Blackmore C, Desnoyers LR (2007). Liver-specific activities of FGF19 require Klotho beta. J Biol Chem.

[54] Park SJ, Shin EJ, Min SS, An J, Li Z (2013). Inactivation of JAK2/STAT3 signaling axis and downregulation of M1 mAChR cause cognitive impairment in klotho mutant mice, a genetic model of aging. Neuropsychopharmacology.

